# Deriving Mechanisms Responsible for the Lack of Correlation between Hypoxia and Acidity in Solid Tumors

**DOI:** 10.1371/journal.pone.0028101

**Published:** 2011-12-09

**Authors:** Hamid R. Molavian, Mohammad Kohandel, Michael Milosevic, Sivabal Sivaloganathan

**Affiliations:** 1 Department of Applied Mathematics, University of Waterloo, Waterloo, Ontario, Canada; 2 Center for Mathematical Medicine, Fields Institute for Research in Mathematical Sciences, Toronto, Ontario, Canada; 3 Radiation Medicine Program, Princess Margaret Hospital, and Department of Radiation Oncology, University of Toronto, Toronto, Ontario, Canada; Karolinska Institutet, Sweden

## Abstract

Hypoxia and acidity are two main microenvironmental factors intimately associated with solid tumors and play critical roles in tumor growth and metastasis. The experimental results of Helmlinger and colleagues (Nature Medicine 3, 177, 1997) provide evidence of a lack of correlation between these factors on the micrometer scale *in vivo* and further show that the distribution of pH and pO_2_ are heterogeneous. Here, using computational simulations, grounded in these experimental results, we show that the lack of correlation between pH and pO_2_ and the heterogeneity in their shapes are related to the heterogeneous concentration of buffers and oxygen in the blood vessels, further amplified by the network of blood vessels and the cell metabolism. We also demonstrate that, although the judicious administration of anti-angiogenesis agents (normalization process) in tumors may lead to recovery of the correlation between hypoxia and acidity, it may not normalize the pH throughout the whole tumor. However, an increase in the buffering capacity inside the blood vessels does appear to increase the extracellular pH throughout the whole tumor. Based on these results, we propose that the application of anti-angiogenic agents and at the same time increasing the buffering capacity of the tumor extracellular environment may be the most efficient way of normalizing the tumor microenvironment. As a by-product of our simulation we show that the recently observed lack of correlation between glucose consumption and hypoxia in cells which rely on respiration is related to the inhomogeneous consumption of glucose to oxygen concentration. We also demonstrate that this lack of correlation in cells which rely on glycolysis could be related to the heterogeneous concentration of oxygen inside the blood vessels.

## Introduction

The tumor microenvironment suffers from hypoxia and acidosis and these microenvironmental stresses play a critical role in tumor cell metabolism, metastasis and the therapeutic response of tumors [1]–[5]. The partial pressure of oxygen (pO_2_) and the pH vary widely over different parts of solid tumors compared to normal tissues [6]. The pH value varies from region to region ranging from highly acidic (6.5) to basic (7.5) and with respect to oxygen content, varies from anoxic areas to very well oxygenated areas [7]–[8]. This heterogeneity, which is a reflection of the complex interaction among the vascular, interstitial and cellular compartments, makes therapeutic intervention difficult since some therapies are effective only over a specific range of pH and/or pO_2_ values [9]. Hence, a better understanding of the mechanisms underlying the heterogeneous distributions of pH and pO_2_, and possible approaches to normalize them, are essential components in the development of efficient treatment strategies. The study of such mechanisms on the micrometer scale, i.e. on the order of the cell size, provides valuable information which can be further extrapolated to the whole tumor.

The experimental results of Helmlinger et al. [10], for the first time, provide information about pH and pO_2_ levels *in vivo*, as functions of distance from blood vessels on a 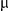
m scale. In this experiment they used the fluorescence imaging microscopy (FRIM) and phosphorescence quenching microscopy (PQM) to simultaneously measure pH and pO_2_ as functions of distance from blood vessels *in vivo* in the dorsal window chamber. Their results demonstrate the spatial heterogeneity in pH and pO_2_ profiles without any specific relationship between them and a correlation between mean values of pH and pO_2_ profiles for a single blood vessel. Based on these results they concluded that (a) there is a lack of correlation between pH and pO_2_ and that (b) the shapes of pH and pO_2_ profiles as functions of distance from adjacent vessels are heterogeneous. However, the mechanisms which trigger this lack of correlation and give rise to these heterogeneous patterns have yet to be uncovered. In addition, some of the observed patterns in their experiments seem to be contrary to our current understanding of cancer cell metabolism. For instance, the presence of hypoxic regions with high pH between blood vessels appears inconsistent with the glycolytic metabolism which would predict low pH in hypoxic regions [10]. Moreover, the observation that the pH curve flattens out at a pH of about 6.9 in normoxic regions between two blood vessels has also not been explained [10].

Recently, Molavian et al. [11] employed the experimental mean values of pH and pO_2_ as functions of distance from a single blood vessel to derive the cell metabolism for the cell line which was used in the experiment of Ref. [10]. In their work they used a mathematical model to calculate the concentration of oxygen, H^+^ and other species for a given cell metabolism. Using this model they were able to find the cell metabolism which is responsible for the observed pH and pO_2_ in Ref. [10]. The obtained metabolism is dominantly respiration in the normoxic region, a combination of glycolysis and respiration in the hypoxic region and it is glycolytic in the anoxic region. Their results indicate that it is necessary to have a large component of respiration in the normoxic region to derive the experimental pH results for the cell line in Ref. [10]. This model could be combined with the experimental results of pH and pO_2_ for a single blood vessel to derive the cell metabolism for other cell lines.

There have been extensive studies on the normalization of the extracellular environment in solid tumors [12]–[14]. It has been suggested that the judicious administration of certain anti-angiogenic agents can normalize the tumor vascular network in a time window, decreasing hypoxia and interstitial fluid pressure [12], [13]. However, to the best of our knowledge, there has been no study of the effects of anti-angiogenesis agents on pH during this normalization window. Some theoretical and experimental studies show that pH levels inside tumors can be increased by augmenting the buffering capacity [15], [16]. However, the effect of varying buffer concentration on the correlation between pH and pO_2_ and on the heterogeneous shapes of pH and pO_2_ profiles in solid tumors has not yet been addressed.

In this paper, we use computer simulations to investigate pH and pO_2_ as functions of distance from blood vessels on the micrometer scale. The simulations are based on our previously described model of tumor metabolism in the neighbourhood of a single blood vessel [11], now modified to include the effect of other adjacent blood vessels. We hypothesise that the three main factors that influence the pO_2_ and pH profiles are: (a) the concentration of nutrients and buffers inside the blood vessels (b) the network of blood vessels, and (c) the cancer cell metabolism. By including an experimental observation, that the concentrations of buffers and oxygen inside blood vessels are heterogeneous, we show through our numerical simulations that the inhomogeneous concentration of species inside the blood vessels is sufficient to describe the observed heterogeneity of the shapes of pH and pO_2_ and the lack of correlation between them. In addition, variations in particular vascular structures and the dependency of the consumption rates of oxygen and glucose on their concentrations can give rise to further complexity in the shapes and relationship of pH and pO_2_ curves. We also propose that the normalization of tumor vessels by the appropriate administration of anti-angiogenesis agents, may result in recovery of the correlation between hypoxia and acidity; however, it may not be able to increase the extracellular pH throughout the whole tumor due to avascular regions present within the tumor. However, an increase in the buffering capacity of the extracellular matrix is an efficient way to enhance pH throughout the tumor. As a result, we propose that the administration of anti-angiogenic agents and at the same time augmentation of the buffering capacity inside the tumor may potentially be the most effective way of normalizing the tumor microenvironment.

## Results and Discussion

### Lack of correlation between pO_2_ and pH

**Figure 1:**
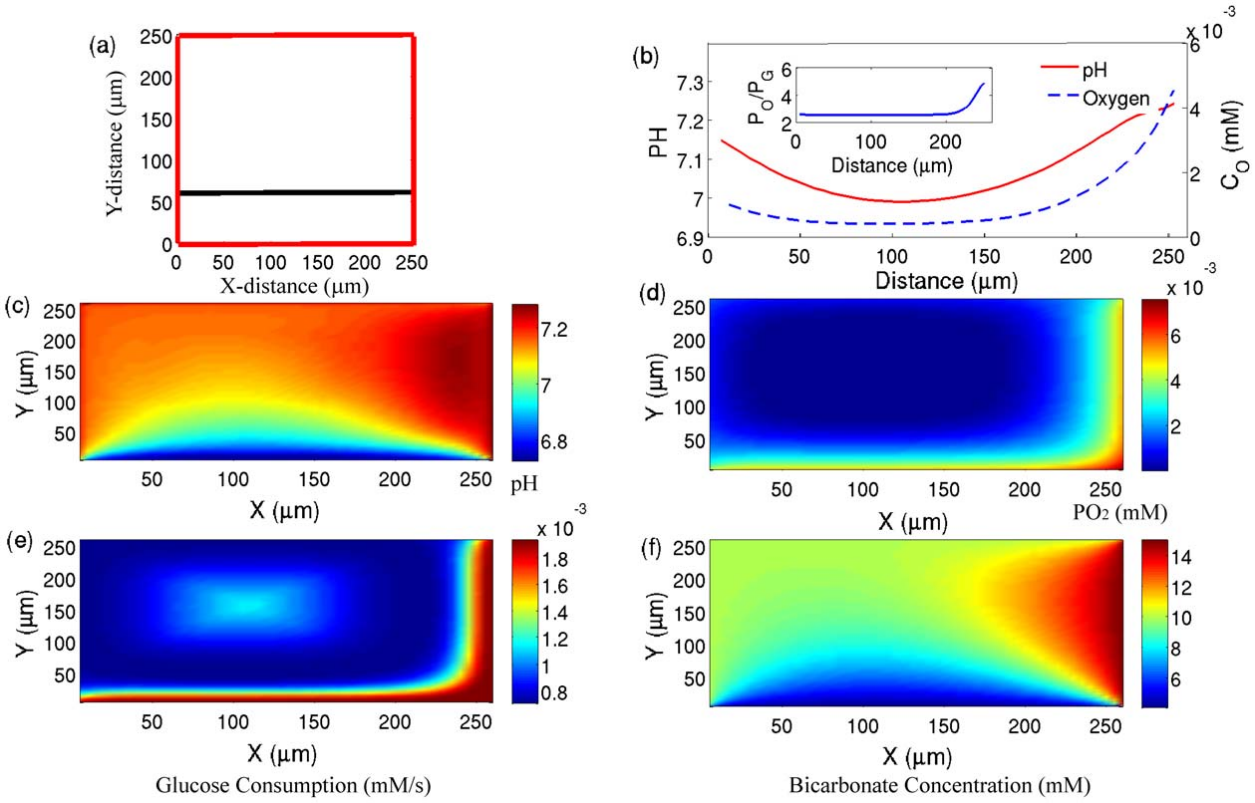
Simulation results for a given arrangement of vessels. a) The structure of blood vessels (red lines) which is similar to Fig. 2e of Ref. [10]. The middle line shows the direction which mimics the experimental measurements. b) The simulated pH (solid line) and pO_2_ (dot-dashed line) as functions of distance along the line which is shown in part a. c) The simulated pH in the XY plane. d) The Simulated pO_2_ in the XY plane. (e) The Simulated glucose consumption in the XY plane. (f) The Simulated bicarbonate concentration in the XY plane.

**Figure 2:**
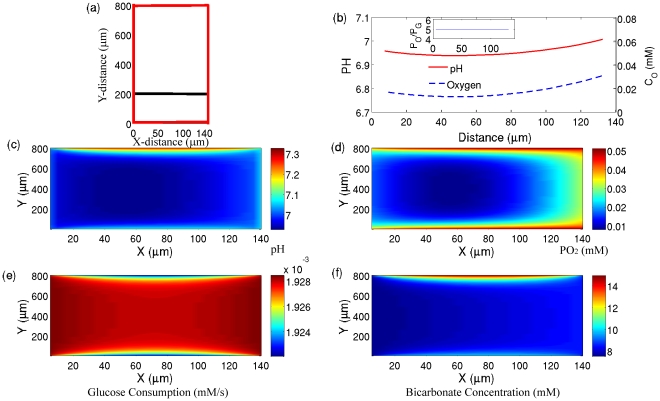
Simulation results for a given arrangement of vessels. a) The structure of blood vessels (red lines) which is similar to Fig. 2i of Ref. [10]. The middle line shows the direction which mimics the experimental measurements. b) The simulated pH (solid line) and pO_2_ (dot-dashed line) as a function of distance along the line which is shown in part a. c) The simulated pH in the XY plane. d) The simulated pO_2_ in the XY plane. (e) The Simulated glucose consumption in the XY plane. (f) The Simulated bicarbonate concentration in the XY plane.

In Figs. 1a and 2a we plot the blood vessel structures (i.e. the arrangement of the vessels) where their sizes are similar to those given in Figs. 2e and 2i (respectively) of the Helmlinger et al. experiments [10]. For these vessels we assume that the concentration of species inside a vessel is constant along the vessel. The values of these concentrations are given in Supplement S1. For oxygen, these values are estimated so that the obtained pO_2_ pattern is consistent with the observed pO_2_ pattern along the measurement experimental line (in the hypoxic region, see Fig. 2 of Ref [10] - for the convenience of readers, this figure is presented in Fig. S3). For example, the concentration of oxygen, in the right and left blood vessels are set up such that they give the experimental values of pH and pO_2_ at the closest points to these sides (at these points the contamination from other blood vessels is negligible). Similarly, the concentrations of H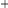, bicarbonate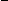 and CO_2_ are adjusted to give the experimentally observed values of pH at both the most left and the most right sides. The only adjustable parameter, which is not directly obtained from the experiment, is the concentration of bicarbonate in the bottom blood vessel (the top blood vessel is far from the measurement line, and hence has a minor effect on pH) - this parameter is set to 10 mM. With this concentration, we are able to derive the pH pattern, in the range of 50–80 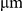 from the bottom blood vessel, which is consistent with Fig. 2f of Ref [10]. Along the experimental measurement line, pO_2_ drops from higher values in the right vessel to lower values in the left vessel; however, it does not follow a hyperbolic shape because at intermediate distances pO_2_ is derived from the bottom vessel and remains constant. It is evident, however, that the pH curve follows a hyperbolic shape and this is due to the fact that (a) pH is approximately the same in the left and right blood vessels, and (b) the production of H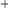 ions by cells, and at the same time the reduction of the buffering capacity, at intermediate distances reduces the pH in the intra-vessel area. The ratio 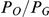 is plotted in the inset of Fig. 1b. This figure shows that except for the cells which are adjacent to the right blood vessel, cells are in the hypoxic region where the consumption mechanism is a combination of respiration and glycolysis. In Figs. 1c and 1d we plot the pH and pO_2_ distribution in the XY plane that is bounded by the four vessels, which is plotted in Fig. 1a, with the aforementioned boundary conditions (see also Supplement S1). The pH for the whole XY plane is in the range 6.75–7.3, which is within the range of experimentally observed pH values in tumors. In Figs. 1e and 1f we respectively plot the glucose consumption and bicarbonate concentration. Comparing the oxygen concentration (Fig. 1d) with the glucose consumption (Fig. 1e) shows that there are regions with high (at the center) and low (around the center) glucose consumptions in the hypoxic area. This originates from the cell metabolism, which shows a higher rate of glucose consumption for lower concentration of oxygen in the hypoxic and anoxic regions. These results demonstrate that the heterogeneous consumption of glucose to oxygen concentration could be one of the parameters responsible for the lack of correlation between glucose uptake and hypoxia observed experimentally [17].

The sizes of the edges of the rectangle in Fig. 2a are chosen such that they simulate the experimental set up of Fig. 2i of Ref. [10]. Again, the concentration of oxygen, bicarbonate, CO_2_ and H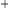 in the left and right blood vessels are fixed such that they are in agreement with the experimental values of pH and pO_2_ right adjacent to the corresponding blood vessels (Supplement S1). In Fig. 2b, we plot pH and pO_2_ as functions of distance along the line of the experimental measurement (a horizontal line 200 µm above the bottom vessel). At this distance the only sources of oxygen for cells are the left and right blood vessels. The results of our simulations for pH and pO_2_ along this line captures the experimental observations of Fig. 2j of Ref. [10]; pO_2_ drops from one vessel to the other vessel and pH remains somehow flat with a small variation of 0.05. To understand this behavior, we observe that 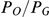 along the line of simulation (which corresponds to the experimental measurement line) is 5 (inset Fig. 2b), hence cells rely predominantly on respiration for the production of ATP. In addition, they produce CO_2_ which acidifies the environment. However, because the blood vessels are close to each other, and the concentration of bicarbonate is high in the whole region (Fig. 2f), the produced H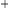 ions are buffered and as a result the pH remains constant. Since the whole region is normoxic, the consumption rate of glucose (Fig. 2e) remains constant and there is a strong correlation between glucose uptake and oxygen concentration.

Figs. 2b and 2d of Ref. [10], show a sharp drop in pH from one of the blood vessels. For example in Fig. 2d, for the left vessel, pH drops from 7.4 to 6.9 within a distance of about 30 µm. These sharp drops show a very high production of H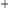 ions in an oxygenated area - these could not be observed in our simulations because in our model the consumption rate of glucose and the production rate of H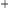 is not high enough to compensate for this sharp drop. One reason for this high production of H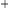 ions could be the presence of cells with high production rate of H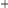 close to the vessel walls. Fig. 2g of Ref. [10], shows two far vessels with almost zero oxygen concentration and pH of around 7.2. Our simulations show that cells close to vessels switch to the glycolytic metabolism and produce H^+^ ions; hence pH drops as a function of distance in this regime (Fig. S1). For large distances (>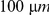) from the vessels, either the lack of glucose or the high concentration of waste products causes pH to saturate to the value of 6.75.

**Figure 3:**
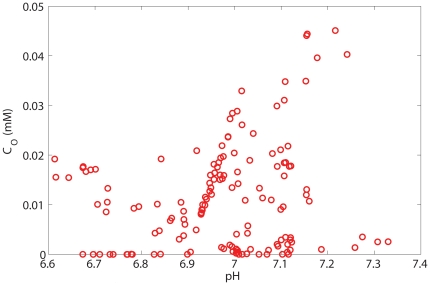
Plot of pH vs. pO_2_ for random points inside the few regions which are bounded by four vessels and with different structure and concentration of species inside the blood vessels.

Figure 3 is a scatterplot of the concentration of Oxygen vs pH for random points inside the rectangular regions (Figs. 1a, 2a and Fig. S1) we described before (correspond to Figs 2e, 2i and 2g of Ref. [10]) plus an extra region with blood vessel structures similar to Fig. 1a but with different concentrations of oxygen and bicarbonate inside the vessels (Supplement S1). We add the data points from this new region to increase the number of random data points and show clearly that there is no correlation between pH and oxygen concentration. This does not reduce the generality of our argument as tumor is comprised of an enormous number of these regions. These regions resemble different parts inside a tumor and we observe non-correlated points similar to the results of Fig. 4 of Ref. [10]. These results show that with a small subset of blood vessel structures, which could be considered to represent different parts of a tumor, the lack of correlation between pH and pO_2_ is very strong. Considering other regions with more complicated blood vessel structures and with different concentrations of species, these will only serve to increase the number of uncorrelated data points and cannot result in a recovery of the correlation between pH and pO_2_.

**Figure 4:**
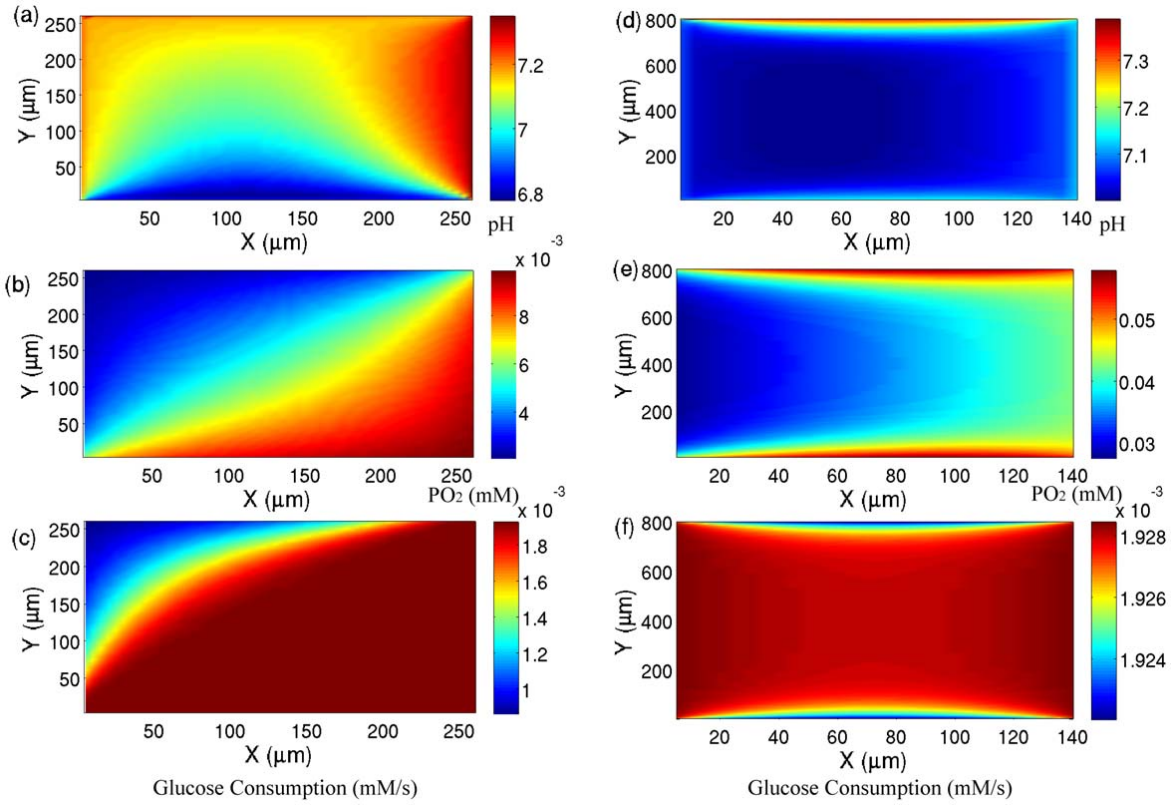
The effect of cell metabolism on pH and pO_2_ and their shapes. The simulated pH of the blood vessel structure of Fig. 1a (a) and 2a (d) and their corresponding pO_2_, respectively (b) and (e) with *r* = 0.05. The glucose consumptions are respectively shown in Figs. (c) and (f).

These results support our hypothesis that the inhomogeneous intravascular concentration of species is a sufficient factor to cause the lack of correlation between pH and pO_2_ and their resultant heterogeneous shapes. Moreover, this abnormality in pH and pO_2_, is amplified by the cell metabolism, as well as by the effects of neighbouring blood vessels. Based on these results we argue that for a chaotic vascular network typical of what is known to exist in most experimental models and in human tumors, which have even less symmetric blood vessel structures than those we have used in our simulations, with different concentrations of nutrients and buffers inside the blood vessels, the pH and pO_2_ pattern will probably be totally uncorrelated and heterogeneous.

### Effects of cell metabolism and the buffering capacity on pO_2_/pH profiles

To understand the importance of different mechanisms on the observed patterns of pH and pO_2_ we investigate the effects of cell metabolism and the buffering capacity on these patterns. In Figs. 4a and 4d we present the pH results for the same blood vessel structures and the same concentrations of species inside the blood vessel (see Figs. 1a and 2a) but this time we set 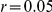. In this region cancer cells rely predominantly on glycolysis instead of respiration. Comparing these results with the patterns of pH in Figs. 1c and 2c, we see indications of some changes in the patterns of pH; however, there is still a lack of correlation between pH and pO_2_ (Figs. 4b and 4e show the corresponding pO_2_ patterns of Figs. 4a and 4d) and furthermore the pattern of pH and pO_2_ are still heterogeneous. Interestingly, for both pH patterns the change in the minimum value of pH is very small. This may sound inconsistent, given that glycolysis directly produces H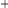 ions and may result in a more acidified environment. However, since we assume that the glucose consumption in normoxia (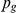) does not depend on 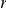, the production of CO_2_ by respiration may produce comparable levels of H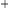 ions. One possible alternative scenario is to increase 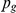 when shifting the metabolism from respiration to glycolysis, which causes a faster drop in pH. This increase in 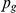 could happen in cell lines with higher rates of glucose consumption compared to the one we have used in our modelling. However, the consumption rate of glucose depends on pH [17] (we have not considered this dependency in our model), and thus a sharp drop in pH may cause a decrease in glucose consumption which is faster than the resultant decrease in the respiration case. Hence, the minimum of pH could not be varied significantly because in addition to the lack of nutrients, the high concentration of waste products and low pH could also reduce the consumption rate of glucose. In Figs. 4c and 4f we plot the glucose consumptions with 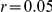 corresponding with the blood vessel structures of Figs. 1a and 2a. Comparing the concentration of oxygen in Fig. 4b with the glucose consumption in Fig. 4c demonstrates that there is no correlation between oxygen concentration and glucose consumption in most of the areas and glucose consumption remains constant. The observed variation in the oxygen concentration is due to the heterogeneity of the oxygen concentration in the blood vessels. The reduction of glucose consumption at the top left of the region is related to the reduction of oxygen concentration in that area of the blood vessels that constitute that edge.

### Implications for therapies

Our results may challenge the prevailing philosophy of trying to normalise the pH throughout the whole tumor by the application of anti-angiogenesis agents. While anti-angiogenesis may reduce hypoxia and increase convection during the normalization window [12], the reduction in hypoxia would result in more cells having access to oxygen and thus may possibly lead to a shift in their metabolism towards respiration. In addition, by the application of anti-angiogenesis agents the concentration of buffers inside the blood vessels may become homogeneous and as a result the correlation between pH and pO_2_ could be recovered. However, the distribution of blood vessels in the normalized tumor is not similar to that in normal tissue as some of the vessels perish as a result of the application of anti-angiogenesis agents and the avascular regions remain without vasculature. This still may not change the minimum of pH observed experimentally as the production of CO_2_ would also tend to acidify the environment and for regions far from the vessels the buffering capacity remains insufficient to normalize the pH. It may be argued that this shift in metabolism goes hand in hand with a reduction in glucose consumption. In this case, the pH and the concentrations of waste products would drop at a lower rate, resulting in an increase in the consumption rates of cells at larger distances. Hence, the effect of anti-angiogenesis agents may result in an increase of pH levels close to blood vessels but may not normalize the pH throughout the whole tumor.

**Figure 5:**
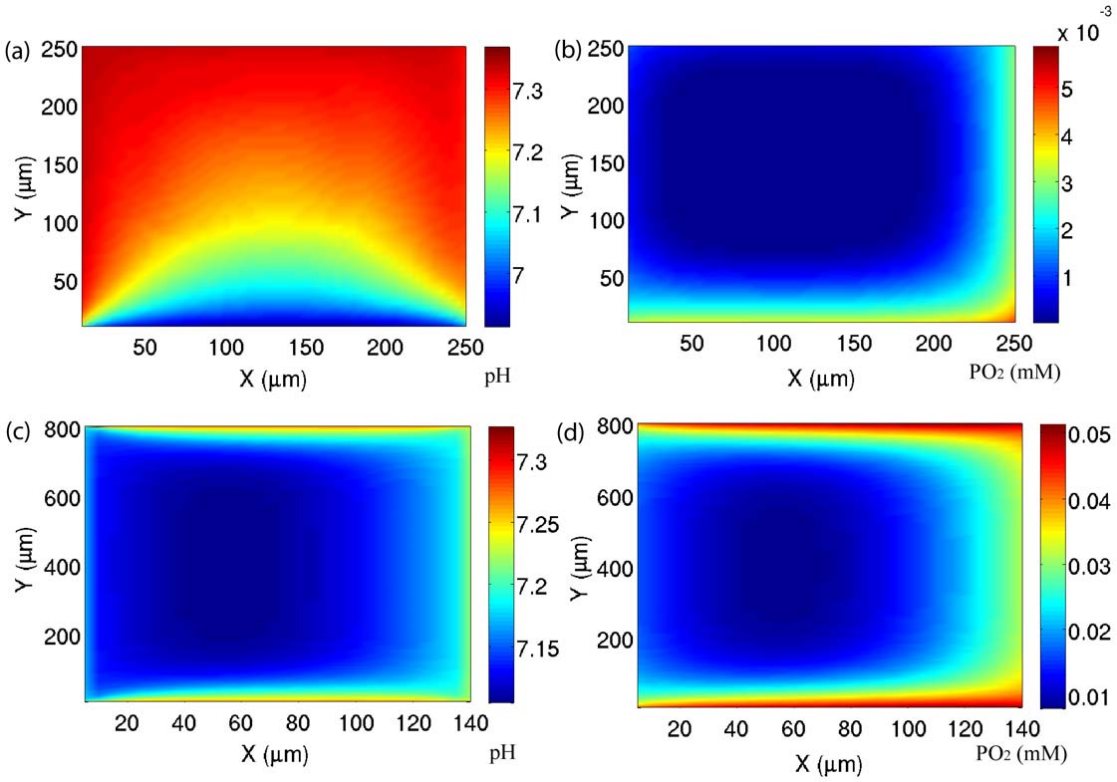
The effect of buffering capacity on the shapes of pH and pO_2_. The simulated pH of blood vessel structure in Fig. 1a (a) and 2a (c) and their corresponding pO_2_, respectively (b) and (d) by increasing the concentration of bicarbonate inside the blood vessels by 50% if C_Bic_<15 mM and leaving the concentration as it is if C_Bic_>15 mM.

Another important factor which directly affects the pH is the buffering capacity of the extracellular environment. This buffering capacity can be affected by adding buffers to the blood vessels and this effect could be taken into account in our model by changing the concentration of bicarbonate inside the blood vessels. In Figs. 5a and 5c we use the same blood vessel structures and the same concentration of species (except for the bicarbonate), see Figs. 1a and 2a, and plot pH as a function of distance - we increase the concentration of bicarbonate by 50% for vessels with C_Bic_<15 mM and leave the concentration of bicarbonates for vessels with C_Bic_≥15 mM unchanged. This models physiological buffers where the pH inside the blood vessels is increased for those vessels with pH less than 7.4 and is left constant for those with pH = 7.4. This increase in the buffering capacity (i.e. the concentration of bicarbonate) enhances the pH throughout the whole region bounded by the vessels. However, it does not necessarily lead to recovery of the correlation between pH and pO_2_ nor to greater homogeneity in the shapes of pH and pO_2_, see Figs. 5b and 5d.

Based on our simulation results, we conclude that the best and most efficient strategy to normalize the tumor microenvironment is to apply the buffers within the normalization window of the anti-angiogenesis agents. This may lead to (a) recovery of the correlation between pH and pO_2_, (b) homogenization of the shapes of pH and pO_2_, and (c) increase pH in avascular regions. These normal patterns are presented in Fig. S2.

It is worth restating that in this work, we have used a specific rectangular geometry with different edge sizes. Although this geometry, to a very good approximation, mimics much of the experimental set up of Ref. [10], the results may be extended to a more complicated network of blood vessels. In fact, for a complex structures of abnormal vasculature, with heterogeneous concentrations of oxygen and buffers inside the vessels, the distribution of pO_2_ is clearly a complicated function of the spatial coordinates. On the other hand, pH does not follow the same pattern as pO_2_ since pH is not only a function of the local pO_2_ but also a function of the local buffering capacity, which depends on the network of blood vessels, and cell metabolism. Hence, in a complicated network of blood vessels, one would expect to observe even more heterogeneous shapes for pH and pO_2_ with again a lack of correlation between them.

**Figure 6:**
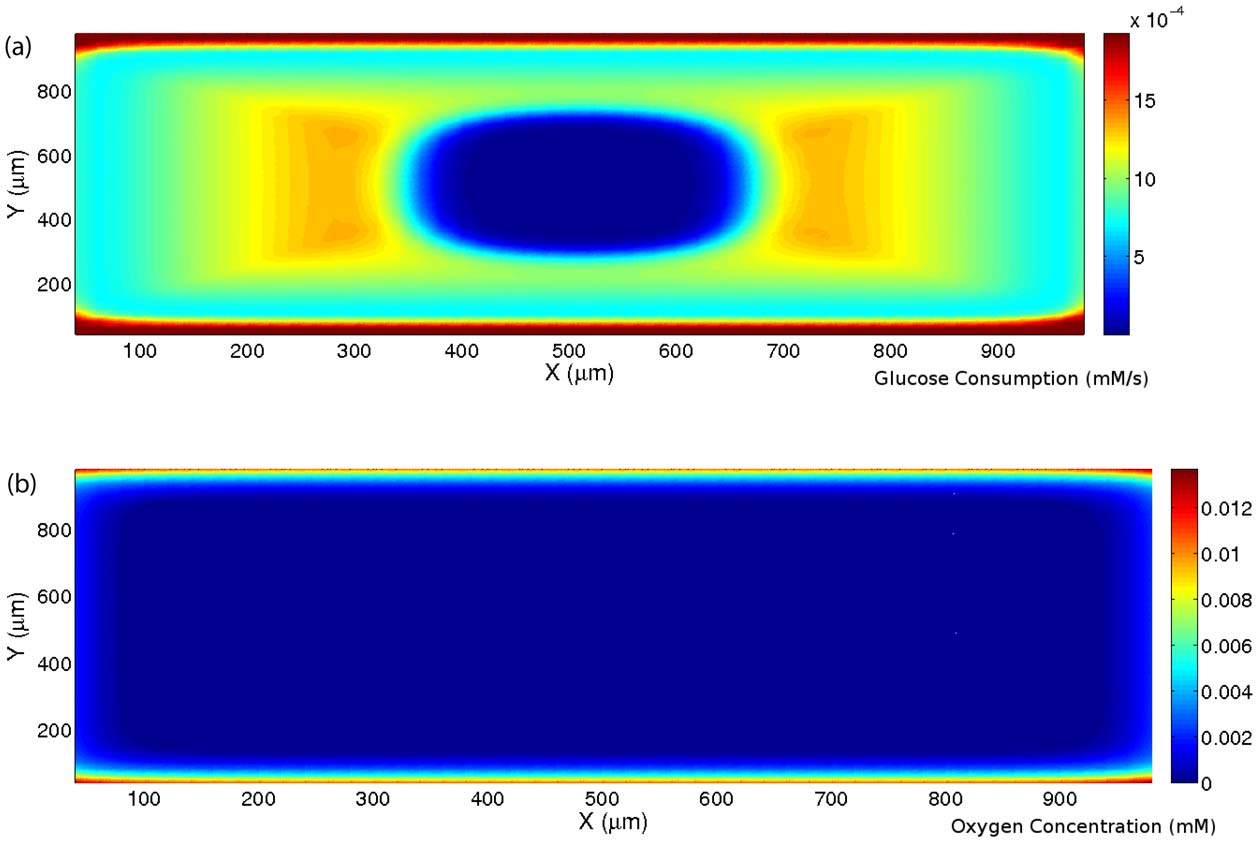
Simulation results for the consumption rate of glucose and oxygen concentration in the XY plane. (a) The consumption rate of glucose, and (b) the concentration of oxygen.

Finally, to show another example of the lack of correlation between oxygen concentration and glucose consumption we consider a rectangular network of four blood vessels with distance 1000 µm with the concentration of species inside the blood vessels as given in Supplement S1. These vessel structures and concentrations are chosen such that they give all different oxygenated regions from anoxic to normoxic. We calculate the concentration of different species and plot the glucose consumption and hypoxia in Fig. 6a and 6b, respectively. These results demonstrate that hypoxic regions with high and very low glucose consumptions are consistent with the experimental observations. Hence the inhomogeneous consumption of glucose as a function of the concentration of oxygen could be the cause of the observed lack of correlation between hypoxia and glucose consumption. The experiments of Ref. [17] could be repeated for the cell line in Ref. [10] to verify our theoretical predictions.

In conclusion, from our simulations we have obtained pH and pO_2_ as functions of distances for a rectangular structure of four blood vessels and investigated the experimentally observed patterns of pH and pO_2_. Based on the experimental observation that the concentration of species inside the blood vessel is heterogeneous we have shown that the lack of correlation between pH and pO_2_ and their heterogeneous shapes are related mainly to the concentration of oxygen and buffers in the blood vessels, and are amplified by cell metabolism and the structure of the blood vessels. Specifically, for regions close to a blood vessel, pH and pO_2_ levels are directly influenced by the concentrations of species inside that vessel and for far regions the whole structure of blood vessels influences the observed levels. As a result, for a chaotic network of blood vessels and heterogeneous concentration of species inside them, we conjecture that pH and pO_2_ still will not follow any specific shapes. We have also demonstrated that for a given glucose consumption in the presence of an excess of glucose and for a certain network of blood vessels, the minimum value of the pH does not vary as a result of a change in cell metabolism from respiration to glycolysis. Based on this, and the fact that cell metabolism depends on pH, we argue that anti-angiogenesis agents may not normalize the pH throughout the whole tumor and may only increase it close to blood vessels. However, our results do suggest that an increase in the buffering capacity within the blood vessels will result in an increase in pH throughout the whole domain of the given network of vessels. Hence, by applying the buffers during the normalization window it is possible to reduce the range of pH and pO_2_ and thus recover homogenous patterns for pH and pO_2_. Our proposal could be verified by applying anti-angiogenesis agents together with the buffers (as in the experimental set up of Ref. [10]) and by measuring the pH and pO_2_ levels as functions of distance. As a by-product of our modeling, we showed that our proposed cell metabolism could explain the lack of correlation between glucose consumption and hypoxia and that this could be also tested by measuring the consumption of glucose and hypoxia in the cell line of Ref. [10].

## Methods

The experimental results of Fig. 2 of Ref. [10], see Fig. S3, show that pH and pO_2_ levels close to the vessel walls are heterogeneous. This is a strong indication that the concentration of species inside the blood vessels (contamination from other vessels is negligible at close distances to a particular vessel wall) are heterogeneous. We hypothesise that this heterogeneity is sufficient to cause the lack of correlation between hypoxia and acidity and force the heterogeneity in the resulting shapes of pH and pO_2_ pattern.

Based on this hypothesis we consider a simple rectangular structure of blood vessels, which to a good approximation describes the experimental set up of Ref. [10]. Although this may seem an oversimplification of the real geometries of tumor vasculature, we argue that if there is a lack of correlation between pH and pO_2_ for this symmetric structure (due to the heterogeneous concentration of species inside the blood vessels; see the results and discussion section), correlation will not be recovered for a more irregular structure and the lack of correlation may even become worse.

By considering this structure of blood vessels, and by changing the size of the edges (Figures 1a and 2a), we derive the different geometrical structures of vessels given in Ref. [10]. We assume that (a) we have a single layer of uniformly distributed cells in the XY plane and that these cells lie within the area bounded by the four blood vessels. (b) Transport of all nutrients and waste products (from metabolic activity) across vessel walls and through the tumor interstitium is by diffusion alone, and that convection is small in comparison, due to the high interstitial fluid pressure. (c) All cells within the region of interest have the same fundamental metabolic balance between aerobic and anerobic metabolism (d) We replace all the buffers present in the extracellular matrix with a single buffer with effective hydration/dehydration rates and effective diffusion constants obtained from Ref. [11]. Finally, all cells within the region of interest are assumed to have the same fundamental metabolic balance between aerobic and anerobic metabolism.

We consider a two-dimensional automaton model with 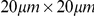 cell size. Following Ref. [11], we consider a minimal model which includes oxygen, glucose, bicarbonate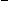, CO_2_, lactate, Na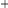, Cl
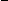
to describe the cell metabolism and relevant mechanisms in the extracellular matrix. We assume that the dominant metabolisms are glycolysis and respiration driven, which are described by the following chemical reactions:
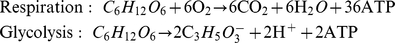
For a respiration dominated metabolism 36ATP are produced by each molecule of glucose. The waste products CO_2_, H
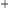
and lactate (C_3_H_5_O_3_ 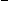) acidify the extracellular matrix whilst, bicarbonate buffers this acidification with the following chemical reaction:
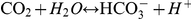
The effective forward and backward rates for this buffer are respectively 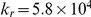 M^−1^ s^−1^ and 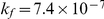 s^−1^, and the diffusion constants for bicarbonate and CO_2_ are respectively 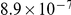 cm^2^/s and 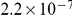 cm^2^/s [18].

We assume that the concentrations of charged and non-charged species in the extracellular environment are in steady state, and are governed by the simple diffusion equation (except Cl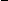) 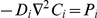, where 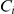 and 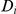 are the concentration and the diffusion constants of species 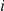, respectively, and 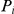 is the production rate of this species (a negative value denotes consumption). For Cl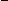, the consumption is zero, hence the electric potential is the only link with the other species. The modified diffusion equation for Cl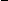, as well as diffusion constants and the boundary conditions for different species are the same as those given in Ref. [11]; see also Supplement S1.

The production (consumption) rates of lactate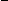, bicarbonate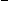, H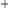 are obtained in terms of bicarbonate and CO_2_ concentrations and, oxygen and glucose consumptions [11]. Na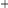 and Cl
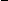
not only contribute to the regulation of intracellular pH by facilitating ion exchange via active membrane transporters, but also the charge neutrality condition in the extracellular environment is also applied and thus the application of any extra forces on the other charged species is avoided. The diffusion equations, with appropriate boundary conditions, are simultaneously solved to obtain the concentration and consumption of these species [11].

Following Ref. [11], we use a cell metabolism in which the consumption rates of glucose and oxygen are respectively 
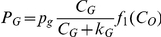
 and 
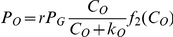
, where 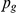
is the maximum consumption rate of glucose under conditions of glucose excess, 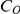
and 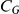 are concentrations of oxygen and glucose respectively, and 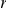 is the ratio between oxygen consumption and glucose consumption in a normoxic area. The functions 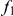 and 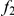 are responsible for the change in cell metabolism from respiration to glycolysis [11]. In the rest of the paper we follow the obtained value for the parameter 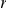 in Ref. [11], namely 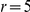 unless another value is explicitly mentioned. This means that in these simulations the cell metabolism is dominated by respiration with a small component of glycolysis in normoxia, followed by a more balanced state of glycolysis and respiration in hypoxia and which becomes primarily glycolysis driven in anoxia. If the value 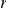 = 0.05 is chosen (see below) this means that the metabolism is dominantly glycolysis even in normoxia (Warburg effect). Our computational model is then used to calculate the pH levels and the concentration of oxygen and other species for a given structure of vessels.

## Supporting Information

Figure S1 **Simulation results for a given arrangement of the vessels.** a) The structure of blood vessels (red lines) which is similar to the Fig. 2g of Ref. [10]. The middle line shows the direction which mimics the experimental measurements. b) The simulated pH (solid line) and pO_2_ (dot-dashed line) as a function of distance along the line which is shown in part a. c) The simulated pH in the XY plane. d) The simulated pO_2_ in the XY plane.
(DOC)

Figure S2 **Simulation results for pH and pO_2_ in the case of dominantly respiration or purely glycolysis.** pH (a and c) and pO_2_ (b and d) for a normal concentration of oxygen and buffers inside the blood vessel. Figs (a) and (b) are the results of cell metabolism with r = 5 (dominantly respiration) and Figs. (c) and (d) are the results of cell metabolism with r = 0.05 (purely glycolysis).
(DOC)

Figure S3 **Measurements of pH and pO_2_.** Adopted (by permission from Macmillan Publishers Ltd: Nature Medicine) from Ref. [10], Helmlinger G, Yuan F, Dellian M, and Jain RK. Interstitial PH and PO2 gradients in solid tumors in vivo: High-resolution measurement reveal a lack of correlation. Nature Medicine 1997;3:177–79.
(DOC)

Supplement S1 **Boundary conditions and values of parameters.**
(DOC)
