# Supplementary material for: Deriving Mechanisms Responsible for the Lack of Correlation between Hypoxia and Acidity in Solid Tumors
Source: PLoS One. 2011 Dec 9;6(12):e28101. doi: 10.1371/journal.pone.0028101 (PMC3235095; doi:10.1371/journal.pone.0028101)
Supplement: Figure S1 — Simulation results for a given arrangement of the vessels. a) The structure of blood vessels (red lines) which is similar to the Fig. 2g of Ref. [10]. The middle line shows the direction which mimics the experimental measurements. b) The simulated pH (solid line) and pO2 (dot-dashed line) as a function of distance along the line which is shown in part a. c) The simulated pH in the XY plane. d) The simulated pO2 in the XY plane. (DOC) [file pone.0028101.s001.doc]

**
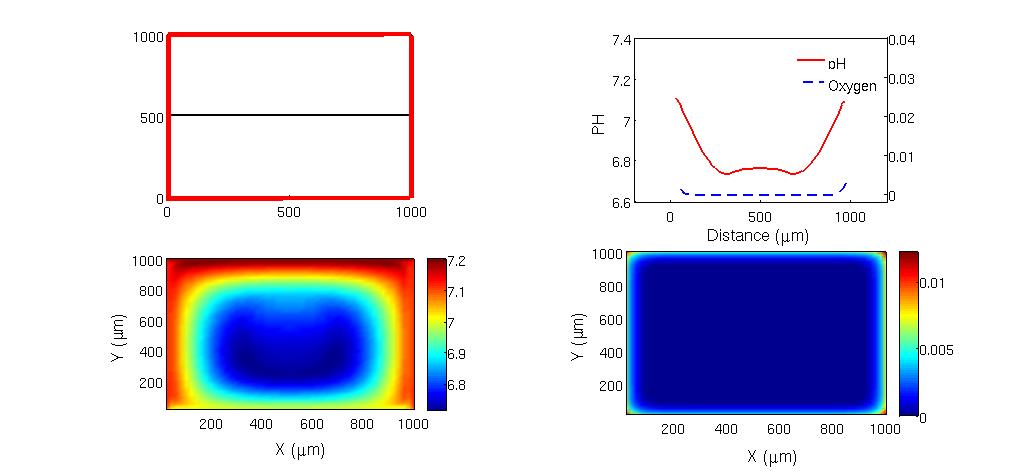
**

**Figure S1**. Simulation results for a given arrangement of the vessels. a) The structure of blood vessels (red lines) which is similar to the Fig. 2g of Ref. [10]. The middle line shows the direction which mimics the experimental measurements. b) The simulated pH (solid line) and pO_2_ (dot-dashed line) as a function of distance along the line which is shown in part a. c) The simulated pH in the XY plane. d) The simulated pO_2_ in the XY plane.
