# Supplementary material for: Deriving Mechanisms Responsible for the Lack of Correlation between Hypoxia and Acidity in Solid Tumors
Source: PLoS One. 2011 Dec 9;6(12):e28101. doi: 10.1371/journal.pone.0028101 (PMC3235095; doi:10.1371/journal.pone.0028101)
Supplement: Figure S2 — Simulation results for pH and pO2 in the case of dominantly respiration or purely glycolysis. pH (a and c) and pO2 (b and d) for a normal concentration of oxygen and buffers inside the blood vessel. Figs (a) and (b) are the results of cell metabolism with r = 5 (dominantly respiration) and Figs. (c) and (d) are the results of cell metabolism with r = 0.05 (purely glycolysis). (DOC) [file pone.0028101.s002.doc]

To illustrate the normalized patterns, we plot pH and pO_2_ respectively in Figs. 2A(a) and 2A(b) for $r=5$ (respiration dominated) and in Figs 2A(c) and 2A(d) for $r=0.05$ (purely glycolysis). For the glycolysis metabolism we assume that cells consume glucose at a constant rate of $p_{g}$. These results are obtained by considering the homogeneous concentration of oxygen and other species inside all four blood vessels. The concentration of oxygen and glucose, bicarbonate$^{-}$ and H$^{+}$ are $6.5\times10^{-2}$mM, $5.9\times10^{-1}$mM, 15mM and $3.98\times10^{-5}$mM respectively and the concentration of other species are fixed to the values given in Supplement S1. These values are set such that all cells have access to enough oxygen and glucose and the ratio between bicarbonate concentration to CO_2_ gives pH=7.4 inside the blood vessel. As clear from these figures, pH and pO_2_ are now fully correlated for both kinds of cell metabolism and have homogenous distribution throughout the region of interest.


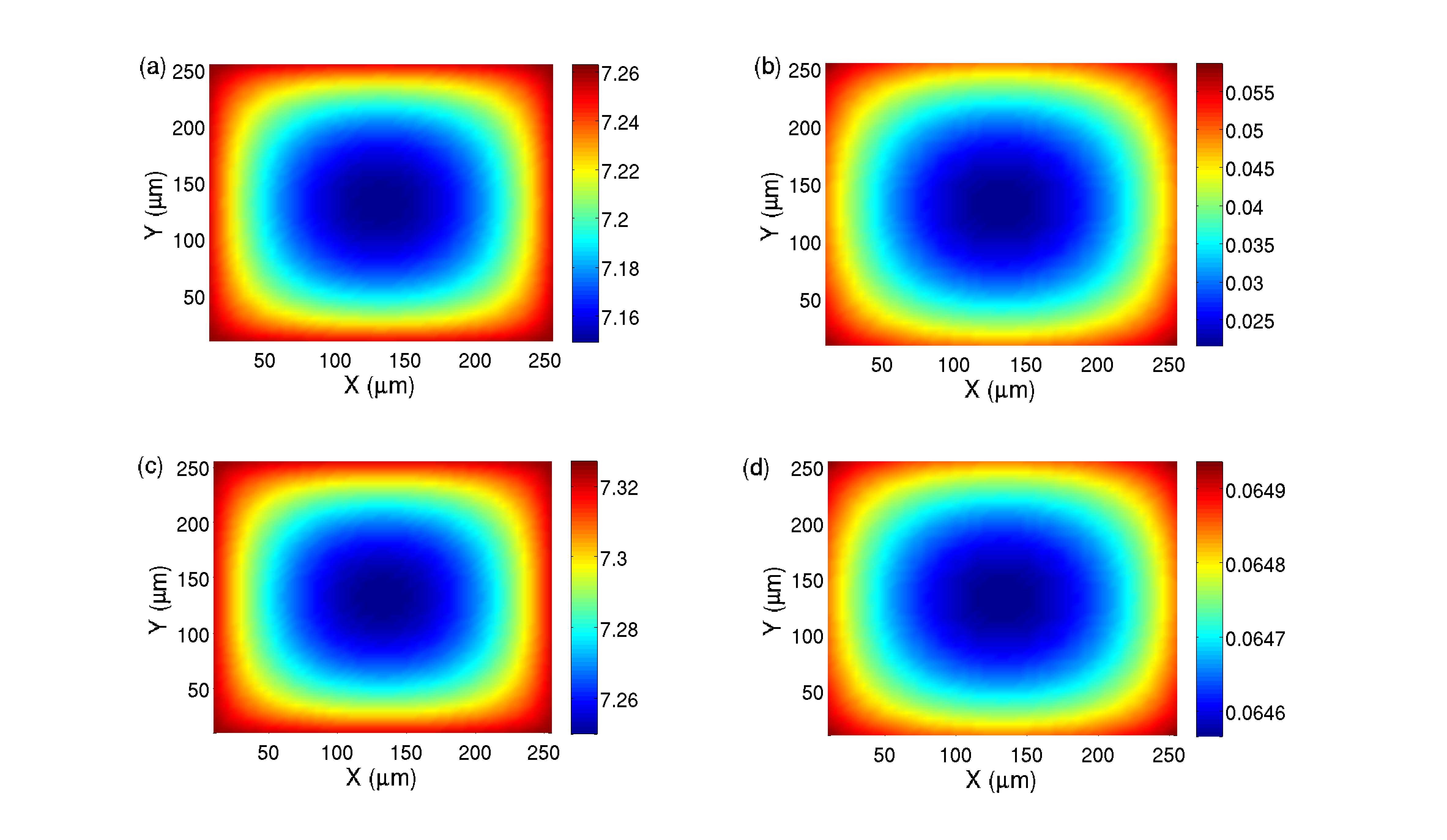


**Figure S2**. Simulation results for pH and pO_2_ in the case of dominantly respiration or purely glycolysis. pH (a and c) and pO_2_ (b and d) for a normal concentration of oxygen and buffers inside the blood vessel. Figs (a) and (b) are the results of cell metabolism with r=5 (dominantly respiration) and Figs. (c) and (d) are the results of cell metabolism with r=0.05 (purely glycolysis).
