# Supplementary material for: Deriving Mechanisms Responsible for the Lack of Correlation between Hypoxia and Acidity in Solid Tumors
Source: PLoS One. 2011 Dec 9;6(12):e28101. doi: 10.1371/journal.pone.0028101 (PMC3235095; doi:10.1371/journal.pone.0028101)
Supplement: Figure S3 — Measurements of pH and pO2. Adopted (by permission from Macmillan Publishers Ltd: Nature Medicine) from Ref. [10], Helmlinger G, Yuan F, Dellian M, and Jain RK. Interstitial PH and PO2 gradients in solid tumors in vivo: High-resolution measurement reveal a lack of correlation. Nature Medicine 1997;3:177–79. (DOC) [file pone.0028101.s003.doc]

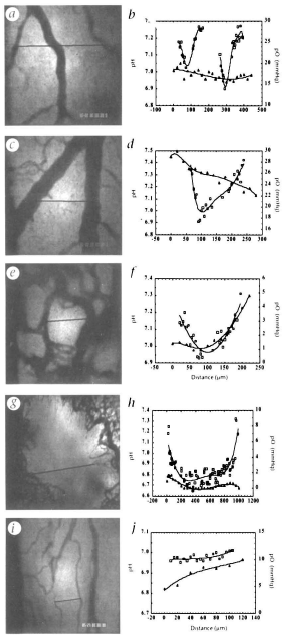


**Figure S3.** Measurements of pH and pO_2_, adopted (by permission from Macmillan Publishers Ltd: Nature Medicine) from Ref. [10], Helmlinger G, Yuan F, Dellian M, and Jain RK. Interstitial PH and PO2 gradients in solid tumors in vivo: High-resolution measurement reveal a lack of correlation. Nature Medicine 1997;3:177-79.
