# Supplementary material for: Deriving Mechanisms Responsible for the Lack of Correlation between Hypoxia and Acidity in Solid Tumors
Source: PLoS One. 2011 Dec 9;6(12):e28101. doi: 10.1371/journal.pone.0028101 (PMC3235095; doi:10.1371/journal.pone.0028101)
Supplement: Supplement S1 — Boundary conditions and values of parameters. (DOC) [file pone.0028101.s004.doc]

**Supplement S1.**

Boundary conditions and values of parameters.

Table 1: The concentrations of Cl$^{-}$, Na$^{+}$, lactate$^{-}$, and CO_2_, in all the blood vessels are respectively $1.05\times10^{2}$mM, $1.22\times10^{2}$mM, 2 mM (1A), 1.5 mM and the rest of the concentrations for different experiments are given in the below tables (in the unit of mM): (A) Fig. 1, (B) Fig. 2, (C) Fig. 3, and (D) Fig. 6.

(A)

|  | Top | Bottom | Left | Right |
| --- | --- | --- | --- | --- |
| H$^{+}$ | $6.61\times10^{-5}$ | $4.36\times10^{-5}$ | $7.59\times10^{-5}$ | $8.51\times10^{-5}$ |
| O2 | $6.5\times10^{-2}$ | $6.5\times10^{-2}$ | $4.5\times10^{-2}$ | $2.5\times10^{-2}$ |
| Glucose | $2.9\times10^{-1}$ | $2.9\times10^{-1}$ | $5.9\times10^{-1}$ | $5.9\times10^{-1}$ |
| Bicarbonate$^{-}$ | 10 | 15 | 8.5 | 7.5 |

(B)

|  | Top | Bottom | Left | Right |
| --- | --- | --- | --- | --- |
| H$^{+}$ | $1.65\times10^{-4}$ | $6.61\times10^{-5}$ | $4.3\times10^{-5}$ | $6.61\times10^{-5}$ |
| O2 | $8\times10^{-3}$ | $2.0\times10^{-3}$ | $8.0\times10^{-3}$ | $2.0\times10^{-3}$ |
| Glucose | $2.9\times10^{-1}$ | $2.9\times10^{-1}$ | $5.9\times10^{-1}$ | $5.9\times10^{-1}$ |
| Bicarbonate$^{-}$ | 4 | 10 | 15 | 10 |

(C)

|  | Top | Bottom | Left | Right |
| --- | --- | --- | --- | --- |
| H$^{+}$ | $8.31\times10^{-5}$ | $5.01\times10^{-5}$ | $6.02\times10^{-5}$ | $6.02\times10^{-5}$ |
| O2 | $2.9\times10^{-2}$ | $2.9\times10^{-2}$ | $3.5\times10^{-2}$ | $3.1\times10^{-2}$ |
| Glucose | $2.9\times10^{-1}$ | $2.9\times10^{-1}$ | $5.9\times10^{-1}$ | $5.9\times10^{-1}$ |
| Bicarbonate$^{-}$ | 8 | 13 | 11 | 11 |

(D)

|  | Top | Bottom | Left | Right |
| --- | --- | --- | --- | --- |
| H$^{+}$ | $1.3\times10^{-4}$ | $1.3\times10^{-4}$ | $4.17\times10^{-5}$ | $4.3\times10^{-5}$ |
| O2 | $6.9\times10^{-2}$ | $6.9\times10^{-2}$ | $2.5\times10^{-2}$ | $2.1\times10^{-2}$ |
| Glucose | $2.9\times10^{-1}$ | $2.9\times10^{-1}$ | $5.9\times10^{-1}$ | $5.9\times10^{-1}$ |
| Bicarbonate$^{-}$ | 5 | 5 | 16 | 15 |

The permeability $\mu$ of each species through a blood vessel and the diffusion constants *D* is given in Table 2. Notice that these permabilities are multiplied by a factor of 50 (the ratio between permeability of normal tissue to cancer tissue) to consider the effect of leakiness. This is a rough approximation, however because of the lack of data for the permeability of species in tumors we used this adjustment. The obtained results (unless for very small values of permeability that gives the zero concentration of species outside the blood vessels) do not depend on the value of these permeabilities.

| Compound | $D$ (cm^2^/s) | Reference | $\mu$ (cm/s) | Reference |
| --- | --- | --- | --- | --- |
| O_2_ | $1.46\times10^{-5}$ | (2A) | $1.5\times10^{-3}$ | (5A) |
| Glucose | $1.10\times10^{-6}$ | (3A) | $1.5\times10^{-3}$ | ** |
| CO_2_ | $8.9\times10^{-7}$ | (11) | $1.5\times10^{-3}$ | ** |
| Glutamine | $1.1\times10^{-6}$ | * | $1.5\times10^{-3}$ | ** |
| Bicarbonate$^{-}$ | $2.2\times10^{-7}$ | (11) | $8.5\times10^{-3}$ | (6A) |
| Lactate$^{-}$ | $1.9\times10^{-6}$ | * | $6\times10^{-2}$ | ** |
| H$^{+}$ | $1.9\times10^{-6}$ | (4A) | $6\times10^{-2}$ | (5A) |
| Cl$^{-}$ | $2.26\times10^{-7}$ | * | $6\times10^{-2}$ | ** |
| Na$^{+}$ | $3.14\times10^{-7}$ | * | $6\times10^{-2}$ | ** |

Table 2: The permeability (through a blood vessel) and diffusion constants for each species. (*) The diffusion constants are calculated by $D_{i}=D_{j}(M_{j}/M_{i})^{0.75}$ (17) where $M_{i}$ is the molecular weight of species$i$. (**) Due to the lack of experimental data the values of the permeabilities of glucose, glutamine, and CO_2_ are chosen to be equal to the value for O_2_, while those of Cl$^{-}$, lactate$^{-}$, and Na$^{+}$are taken to be equal to that of H$^{+}$.

**Supplement References**

1. Bell G, Davidson JN, Scarborough H. Textbook of Physiology and Biochemistry. Seventh Edition Baltimore: The Williams and Wilkins Company; 1968. P. 738.
2. Nicholas MG and Foster TH. Oxygen diffusion and reaction kinetics in the photodynamic therapy of multicell tumor spheroid. Phys. Mod. Biol. 1994;39:2161-21.
3. Casciari JJ, Sotirchos SV, and Sutherland RM. Glucose diffusity in Multicellular Tumor Spheroids. Cancer Research 1988;48:3905-9.
4. Fatt I,Giasson CJ, and Mueller TD. Non-Steady-State Diffusion in a Multilayered Tissue Initiated by Manipulation of Chemical Activity at the Boundaries. Biophysical Journal 1998;74:475-86.
5. Crone C, and Levitt DG. Capillary permeability to small solutes. In: Handbook of Physiology: A Critical, Comprehensive presentation of physiology knowledge and concepts, Section 2:The Cardiovascular System Vol IV:Microcirculation, part 1 (Renkin EM, and Michel CC, eds) P 414 and PP. 434-7. Bethesda, ML: American Physiology Socity.
6. Chan YL, Malnic G, Giebisch G. Passive driving forces of proximal tubular fluid and bicarbonate transport: gradient dependence of H^+^ secretion. Am J Physiol Renal Physiol 1983;245:F622-F633.
